# The Medical Department of Johns Hopkins University

**Published:** 1907-09-07

**Authors:** 


### THE MEDICAL DEPARTMENT OF JOHNS HOPKINS UNIVERSITY.

The undergraduate department is planned for the training of those who have received a liberal education. A collegiate degree in arts or science from a recognised university or college is necessary for admission. In addition to this the applicant must have a reading knowledge of French and German, with an adequate training in the preliminary scientific branches, such as chemistry, physics and biology. The course extends over a period of four years, the term being eight months—from October 1 to June 1. The students proceed to the degree of M.D.

Throughout the course special emphasis is laid on practical work in the laboratories and the hospital, which is regarded as an integral part of the medical school. In the first two years of the course anatomy, physiology, physiological chemistry, pharmacology, and toxicology, pathology and bacteriology are taken up. In the final years the hospital—both the wards and the out-patient department—is the class-room. Didactic lectures are few, and practical instruction is given to small groups as far as possible. The fourth-year students spend a large part of their time working in the wards as clerks and dressers. Beginning with the session of 1907 some elective courses will be given in the final year, the student having certain freedom of choice in deciding which of the special subjects he will take.

The courses for graduates are of two kinds: (1) Special and (2) University courses.

(1) The Special courses are usually given during May and June of each year to limited classes. They embrace courses in medicine (clinical work, classes in physical diagnosis and clinical microscopy), operative surgery on animals, surgical pathology, pediatrics, orthopædic surgery, genito-urinary surgery, *x*-ray diagnosis, gynæcological pathology, cystoscopy, ophthalmology, etc. These courses are practical as far as possible, and are designed specially for general practitioners.

(2) The University courses differ from these in that they are given throughout the session. These are of two kinds—elementary and advanced. The former are much the same as the work being done by the undergraduates, and are usually for periods of three or six months. In all of these the number who can be admitted is necessarily limited, and applicants must satisfy the head of the department of their fitness to profit by the work. Thus elementary courses are given in medicine, pathology ,bacteriology, physiology, gross anatomy, neurological anatomy, and physiological chemistry. For the advanced and research courses only those physicians are accepted who are prepared to undertake special studies and to spend sufficient time to make their admission worth while. Thus, in medicine, work may be done in the various laboratories of the medical clinic, *e.g.* the general clinical laboratory, the biological laboratory which investigates biological methods of diagnosis and treatment, the physiological laboratory, and the bio-chemical laboratory. In the Phipp's Dispensary there is a laboratory for the study of the problems of tuberculosis. In the pathological department courses on special work, such as the pathology of the organs of special secretion, are given. A limited number are admitted for research work. In the anatomical and physiological laboratories a few selected men are admitted for advanced work, as, for example, in the anatomy of the nervous system, embryology and physiology. A similar course is available in physiological chemistry. In all of these the fitness of the applicant is decided by the head of the department.

